# An Analysis of Online Evaluations on a Physician Rating Website: Evidence From a German Public Reporting Instrument

**DOI:** 10.2196/jmir.2655

**Published:** 2013-08-06

**Authors:** Martin Emmert, Florian Meier

**Affiliations:** ^1^Institute of Management (IFM)School of Business and EconomicsFriedrich-Alexander-University Erlangen-NurembergNurembergGermany

**Keywords:** physician rating website, public reporting, quality of care, Internet, patient satisfaction

## Abstract

**Background:**

Physician rating websites (PRW) have been gaining in popularity among patients who are seeking a physician. However, little evidence is available on the number, distribution, or trend of evaluations on PRWs. Furthermore, there is no published evidence available that analyzes the characteristics of the patients who provide ratings on PRWs.

**Objective:**

The objective of the study was to analyze all physician evaluations that were posted on the German PRW, jameda, in 2012.

**Methods:**

Data from the German PRW, jameda, from 2012 were analyzed and contained 127,192 ratings of 53,585 physicians from 107,148 patients. Information included medical specialty and gender of the physician, age, gender, and health insurance status of the patient, as well as the results of the physician ratings. Statistical analysis was carried out using the median test and Kendall Tau-b test.

**Results:**

Thirty-seven percent of all German physicians were rated on jameda in 2012. Nearly half of those physicians were rated once, and less than 2% were rated more than ten times (mean number of ratings 2.37, SD 3.17). About one third of all rated physicians were female. Rating patients were mostly female (60%), between 30-50 years (51%) and covered by Statutory Health Insurance (83%). A mean of 1.19 evaluations per patient could be calculated (SD 0.778). Most of the rated medical specialties were orthopedists, dermatologists, and gynecologists. Two thirds of all ratings could be assigned to the best category, “very good”. Female physicians had significantly better ratings than did their male colleagues (*P*<.001). Additionally, significant rating differences existed between medical specialties (*P*<.001). It could further be shown that older patients gave better ratings than did their younger counterparts (*P*<.001). The same was true for patients covered by private health insurance; they gave more favorable evaluations than did patients covered by statutory health insurance (*P*<.001). No significant rating differences could be detected between female and male patients (*P*=.505). The likelihood of a good rating was shown to increase with a rising number of both physician and patient ratings.

**Conclusions:**

Our findings are mostly in line with those published for PRWs from the United States. It could be shown that most of the ratings were positive, and differences existed regarding sociodemographic characteristics of both physicians and patients. An increase in the usage of PRWs might contribute to reducing the lack of publicly available information on physician quality. However, it remains unclear whether PRWs have the potential to reflect the quality of care offered by individual health care providers. Further research should assess in more detail the motivation of patients who rate their physicians online.

## Introduction

In many health care systems, quality of care improvement strategies have been implemented over the last few years [1]; nevertheless, quality deficits still remain [2-4]. Several studies have further shown remarkable variability in quality of care across health care providers [1,5-7]. However, patients are not likely to be generally aware of existing quality differences [8,9]. One reason for this is the limited amount of publicly reported information on the quality of health care providers [10].

It has become a major challenge to remedy this deficiency by improving transparency about the quality of health care providers [10,11]. This is supposed to increase overall quality by steering patients to better performing health care providers [12,13] and by motivating providers to make quality improvements [9,14]. Therefore, public reporting (PR) instruments have been put in place in many countries [15-22]. These instruments generally assess the quality of care by measuring adherence to clinical guidelines and by providing additional structural information [11]. However, patients have been slow to take advantage of these comparative reports in making their health care provider choices [9]. Possible reasons for this might be found in the fact that patients are not aware of the information, do not understand it, do not believe it, or are unwilling or unable to use the information provided [23].

The newest trend in the PR movement is the use of physician rating websites (PRWs) [24]. The primary objective of these websites lies in rating and discussing physician quality online by using user-generated data [25,26]. Although the usefulness of PRWs has been seen critically from a scientific point of view [24], their popularity among patients has been increasing [24,27,28]. In contrast to traditional PR instruments, PRWs might have the advantage that the information can be more easily understood by patients. While traditional instruments report on measures such as the administration of beta blockers or angiotensin-converting enzyme inhibitors, which require a higher level of clinical knowledge than most patients have [8], PRWs concentrate on measuring patient satisfaction [24].

Although there is a vast amount of evidence regarding traditional PR instruments, little research has addressed PRWs [25]. A recently conducted systematic review has identified 9 articles published in peer-reviewed journals [25]. In them, the number, distribution, and trend of the evaluations on PRWs were investigated [11,27-34]. Most of the investigations evaluated ratings for a (non)random sample of physicians, while 1 study assessed over 386,000 national ratings from 2005 to 2010 from the US PRW, RateMDs. Furthermore, there is no published evidence available that analyzes the characteristics of the patients who provide ratings.

In this context, this paper adds to the literature by presenting an analysis of all physician evaluations posted on the German PRW, jameda, in 2012. Thereby, we provide descriptive analysis of (1) both physician and patient characteristics, and (2) the number, distribution, and results of the ratings. Analytical analyses were applied to assess (3) the impact of physician and patient characteristics on the overall performance measure, and (4) the correlation between the number of ratings per patient/physician and the overall performance.

## Methods

### Analysis of Jameda

This paper presents an analysis of all 127,192 physician evaluations that were posted on the German PRW, jameda, in 2012. In total, 107,148 patients completed evaluations on 53,585 physicians. The dataset contained the following information: the medical specialty and gender of the physician, as well as the gender, age, and health insurance status of the patient. Additionally, the results of the physician ratings for all mandatory and optional questions were included. The mandatory physician rating system on jameda consists of 5 questions, rated according to the grading system in German schools on a 1-6 scale (1=very good; 2=good; 3=satisfactory; 4 =fair; 5=deficient; and 6=insufficient) [35]. These relate to (Q1) satisfaction with the treatment offered by the physician, (Q2) education about the illness and treatment, (Q3) the relationship of trust with the physician, (Q4) the time the physician spent on the patient´s concerns, and (Q5) the friendliness of the physician. A mean score (“overall performance”) is calculated, based on the results of these 5 questions. Beyond that, a narrative commentary has to be given and 13 optional questions are available for answering (these are not addressed in this paper) [36].

We focused on jameda because it is likely to play the most significant role in the German PRW movement for the following reasons: (1) from a patient’s perspective, jameda is the PRW to which a patient is most likely to be referred [24,31], (2) jameda is ranked highest in traffic among German PRWs [34], and (3) among German PRWs, jameda has been shown to contain the largest number of ratings, so far [37].

### Statistical Analysis

All statistical analyses were conducted using SPSS 21.0 (SPSS for Windows, version 21.0). The median test was used for nonparametric data of groups with different distributions. The Kendall Tau-b test was used to analyze specific correlations. Differences were considered to be significant if *P*<.05 and highly significant if *P*<.001.

## Results

### Number and Distribution of Ratings

In total, 127,192 ratings of 53,585 physicians from 107,148 patients were posted on the PRW, jameda, in 2012. The German outpatient sector consists of approximately 146,000 physicians [38]; thus, 37% were rated in 2012. As displayed in Table 1, about one third of all rated physicians were female (34.1%). The rating patients were mostly female (60%), between 30-50 years (51%), and covered by Statutory Health Insurance (83%).

The distribution of ratings demonstrates that nearly half of the physicians were rated once and less than 2% were rated more than ten times (see Table 2). Thereby, rated physicians had a mean of 2.37 individual ratings (SD 3.169, range 1-159). It could further be shown that 88% of the patients left a single rating and 12% of them left between two and five ratings. This leads to an average of 1.19 rated physicians per patient (SD 0.778, range 1-153).

If the ratings are analyzed according to the medical specialty of the physicians in absolute terms, family physician/general practitioner, internist, and gynecologist were rated most often (13,466, 8709, and 6410, respectively) (see Table 3; [38,39]). In contrast, laboratory specialist, nuclear medicine, and child and youth psychotherapist were rated least frequently (13, 136, and 166, respectively). The distribution of ratings in relative terms, compared to the national physician composition, shows that the most rated medical specialties were orthopedists, dermatologists, and gynecologists (59.20%, 58.90%, and 56.90%, respectively). In contrast, the least frequently rated medical specialties were radiologists, anesthetists, and laboratory specialists (10.40%, 7.90%, and 2.10%, respectively).

**Table 1:** Number and distribution of ratings on jameda (gender, age, insurance).

Characteristics		Absolute	%	%, cum
**Gender—Physician**				
	Female	18,284	34.1	34.1
	Male	35,301	65.9	100.0
	Total	53,585	100.0	
**Gender—Patient**				
	Female	48,171	45.0	45.0
	Male	31,809	29.7	74.7
	n.a.	27,168	25.4	100.0
	Total	107,148	100.0	
**Age—Patient**				
	<30	13,639	12.7	12.7
	30-50	38,608	36.0	48.8
	50+	23,676	22.1	70.9
	n.a.	31,225	29.1	100.0
	Total	107,148	100.0	
**Health insurance—Patient**				
	Statutory health insurance	64,986	60.7	60.7
	Private health insurance	13,402	12.5	73.2
	n.a.	28,760	26.8	100.0
	Total	107,148	100.0	

**Table 2:** Number and distribution of ratings on jameda (physicians and patients).

	Number of ratings	Absolute	%	%, cum
**Physicians**				
	1	26,615	49.7	49.7
	2-5	23,430	43.7	93.4
	6-10	2,664	5.0	98.4
	11-50	849	1.6	99.9
	51+	27	0.1	100.0
	Total	53,585	100.0	
**Patients**				
	1	94,099	87.8	87.8
	2-5	12,702	11.9	99.7
	6-10	329	0.3	100.0
	11-51	17	0.0	100.0
	51+	1	0.0	100.0
	Total	107.148	100.0	

**Table 3:** Number and distribution of ratings according to medical specialty.

Medical specialty	Rated physicians in absolute terms (%)	Number of physicians in Germany^a^	Rated physicians in relative terms (%)
Orthopedist	3677 (6.9)	6206	59.2
Dermatologist (incl venereologist)	2445 (4.6)	4154	58.9
Gynecologist	6410 (12.0)	11,256	56.9
Oral maxillo-facial surgeon	634 (1.2)	1,122	56.5
Neurosurgeon	337 (0.6)	608	55.4
ENT specialist, otorhinolaryngologist	2304 (4.3)	4301	53.6
Urologist	1545 (2.9)	3030	51.0
Neurologist/Psychiatrist	2685 (5.0)	5775	46.5
Pediatrician	2957 (5.5)	6866	43.1
Medical practitioner without specialization	1697 (3.2)	4252	39.9
Ophthalmologist	2253 (4.2)	5796	38.9
Internist	8709 (16.3)	23,198	37.5
Family physician/General practitioner	13,466 (25.1)	36,196	37.2
Nuclear medicine	136 (0.3)	698	19.5
Child and youth psychotherapist	166 (0.3)	922	18.0
Others	3432 (6.4)	23,561^b,c^	14.6
Radiologist (incl radiotherapist)	421 (0.8)	4,029	10.4
Anesthetist	298 (0.6)	3796	7.9
Laboratory specialist	13 (0.0)	623	2.1
Total	53,585 (100.0)	146,389	36.6

^a^If not other than [38].

^b^According to [39].

^c^Others (eg, surgeon, psychotherapist, pathologist, pneumologist).

### Evaluations

Table 4 shows the evaluation results of all 53,585 rated physicians (as they are displayed on the website). It can be shown that two thirds of all evaluations were assigned to the best rating category, “very good”. An additional 13% of patients rated their experience with the physician as “good”. Three percent of the physicians were rated with the worst score, “insufficient” in their overall performance. The median result of all questions was “very good”, while the mean varied between 1.68 for question 5 (friendliness of the physician) and 1.85 for question 3 (relationship of trust with the physician).

An analysis was performed to ascertain whether differences in the rating of a physician, regarding both the physician (ie, gender and medical specialty) and the patient characteristics (ie, gender, age, and health insurance) could be determined. The results are displayed in Table 5. They show that female physicians were rated better than their male colleagues and that the difference is statistically significant (the percentage of rated physicians below median is 61% for female and 59% for male physicians; *P*<.001). Furthermore, significant rating differences between medical specialties could be demonstrated (*P*<.001). The best rated medical specialties were laboratory specialists, anesthetists, medical practitioner without specialization, and family physician/general practitioner (85%, 76%, 74%, and 70% below median, respectively). The lowest ratings were given to neurologist/psychiatrist, ophthalmologist, orthopedist, and dermatologist (including venereologist) (47%, 45%, 35%, and 35% below median, respectively).

With respect to patient characteristics, no significant rating differences between female and male patients could be detected (percentage below median is 59% in each group; *P*=.505). However, it could be shown that older patients gave better ratings than did their younger counterparts (*P*<.001). Additionally, patients covered by private health insurance gave more favorable evaluations than did patients covered by statutory health insurance (*P*<.001).

**Figure 1:**
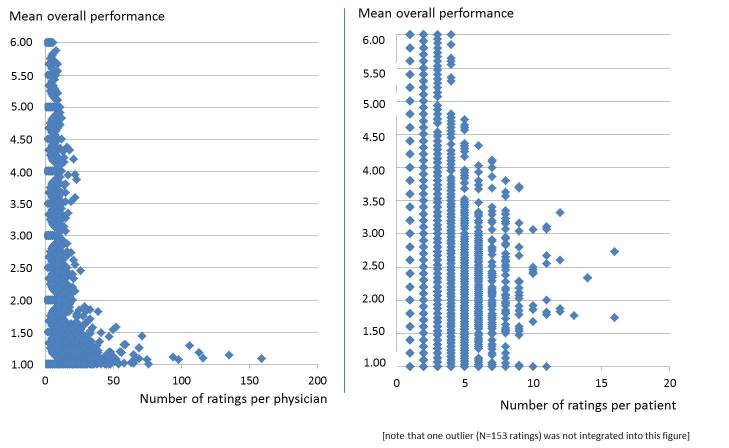
Scatterplot (bivariate); the number of ratings per physician (left)/patient (right) with the mean overall performance for a rated physician.

Next, the correlation between the mean overall performance of a physician and the number of ratings per physician was addressed. As displayed in Figure 1, the total performance range can be observed for physicians with a low number of ratings. By contrast, physicians who received a higher number of ratings were shown to have better ratings (eg, all physicians with more than 60 ratings were rated as “very good”). As a result, the correlation between the mean overall performance of a physician and the number of ratings per physician could be shown to be statistically significant (Kendall Tau-b=0.193, *P*<.001). This is also true for all five mandatory questions (*P*<.001; data not presented here). We further investigated to find out whether similar results could be detected for the number of ratings per patient compared to the mean overall performance given by this patient. The result is displayed in Figure 1 and shows a similar correlation (Kendall Tau-b=0.178, *P*<.001).

**Table 4:** Evaluation results of all rated physicians on jameda.

		Overall performance	Q1^b^	Q2^c^	Q3^d^	Q4^e^	Q5^f^
**Performance range** ^a^ **, n (%)**						
	1	35,227 (65.7)	35,030 (65.4)	33,345 (62.2)	34,665 (64.7)	34,331 (64.1)	36,708 (68.5)
	2	7170 (13.4)	7302 (13.6)	8660 (16.2)	6748 (12.6)	7535 (14.1)	7313 (13.6)
	3	4694 (8.8)	4876 (9.1)	5019 (9.4)	5077 (9.5)	5075 (9.5)	4305 (8.0)
	4	2615 (4.9)	2312 (4.3)	2584 (4.8)	2350 (4.4)	2512 (4.7)	2201 (4.1)
	5	2429 (4.5)	2000 (3.7)	1988 (3.7)	1972 (3.7)	1992 (3.7)	1461 (2.7)
	6	1450 (2.7)	2065 (3.9)	1988 (3.7)	2773 (5.2)	2139 (4.0)	1597 (3.0)
Total		53,585 (100.0)	53,585 (100.0)	53,584 (100.0)	53,585 (100.0)	53,584 (100.0)	53,585 (100.0)
Mean		1.77	1.79	1.83	1.85	1.82	1.68
Median		1.00	1.00	1.00	1.00	1.00	1.00
SD		1.32	1.35	1.34	1.43	1.37	1.24
Minimum		1.00	1.00	1.00	1.00	1.00	1.00
Maximum		6.00	6.00	6.00	6.00	6.00	6.00

^a^German school based rating system (1=very good; 2=good; 3=satisfactory; 4=fair; 5=deficient; 6=insufficient).

^b^Q1: satisfaction with the treatment by the physician.

^c^Q2: education about the illness and treatment.

^d^Q3: relationship of trust with the physician.

^e^Q4: time the physician spent for the patient´s concerns.

^f^Q5: friendliness of the physician.

**Table 5:** Ratings differences regarding physician and patient characteristics.

Characteristics		N	> Median	≤Median	Percentage below median (%)	*P* value^a^
**Gender—Physician**						<.001
	Female	18,284	7168	11,116	61	
	Male	35,301	14,583	20,718	59	
**Medical specialty—Physician**						<.001
	Laboratory specialist	13	2	11	85	
	Anesthetist	298	72	226	76	
	Medical practitioner without specialization	1697	433	1264	74	
	Family physician/General practitioner	13,466	4081	9385	70	
	Oral maxillo-facial surgeon	634	197	437	69	
	Internist	8709	2889	5820	67	
	Nuclear medicine	136	47	89	65	
	Urologist	1545	562	983	64	
	Neurosurgeon	337	124	213	63	
	Pediatrician	2957	1180	1777	60	
	Radiologist (incl radiotherapist)	421	174	247	59	
	Gynecologist	6410	2662	3748	58	
	Others	3432	1504	1928	56	
	Child and youth psychotherapist	166	78	88	53	
	ENT specialist, otorhinolaryngologist	2304	1108	1196	52	
	Neurologist/Psychiatrist	2685	1424	1261	47	
	Ophthalmologist	2253	1241	1012	45	
	Orthopedist	3677	2380	1297	35	
	Dermatologist (incl venereologist)	2445	1593	852	35	
**Gender—Patient**						.505
	Female	48,171	19,989	28,182	59	
	Male	31,809	13,124	18,685	59	
**Age—Patient**						<.001
	<30	13,639	6697	6942	51	
	30-50	38,608	16,064	22,544	58	
	50+	23,676	8542	15,134	64	
**Health Insurance—Patient**						<.001
	Statutory Health Insurance	64,986	28,309	36,677	56	
	Private Health Insurance	13,402	4523	8879	66	

^a^Median test.

## Discussion

### Principal Findings

In this section, the results obtained in this investigation are compared to published studies, mostly from the United States. The evidence from this investigation shows that 37% of physicians in the German outpatient sector were rated on jameda in 2012. This number exceeded those from previously published international studies. For example, Gao and colleagues showed that 16% of US physicians received an online review on RateMDs in the period between 2005 and 2010 [27]. Lagu et al reported that out of 300 Boston physicians, 27% of them had been rated [11], while Mostaghimi et al calculated percentages of between 0.4% and 21% for a sample of 250 randomly selected internal medicine physicians [33]. In a sample of 500 randomly selected US urologists, the percentages varied between 0.4% and 53.6% [40]. Published results for German PRWs reported percentages of between 3.36% and 25.78% in 2009 [31] and between 3% and 28% in 2012 [34]. However, it is worth mentioning here that direct comparison is difficult due to the fact that data from one year was analyzed in this investigation, whereas most studies use ratings for a sample of physicians without including any time constraints.

It could also be shown that rated physicians had a mean of 2.37 individual ratings (SD 3.169, range 1-159). Published results for the US PRW, RateMDs, were quite similar and were reported to be 2.7 [30], respectively 3.2 [27]. More recent US studies determined numbers of 2.35 [11] and 2.4 [40], while results for German PRWs were reported to be between 1.1 and 3.9 [34]. The number decreases to 0.87 when regarding all rated physicians from the German outpatient sector in 2012. This is slightly higher than the results obtained by Lagu and colleagues (mean 0.63) [11].

Nearly half of the physicians were rated only once, and 44% received between 2 and 5 ratings in this study. Less than 2% were rated more than 10 times and 0.1% more than 50 times. These numbers are in line with the results obtained by analyzing the ratings provided for 2010 on RateMDs. In that case, half of the physicians had a single rating and the percentage of physicians with 5 or more ratings was 12.50% [27]. Of 250 randomly selected physicians in Boston, 50 physicians (20%) had between 1 and 4 reviews on Healthgrades, 13 physicians (5.2%) on RateMDs, and 1 physician (0.4%) on Wellness. Only 3 physicians had more than 5 reviews on any of the ratings sites [33].

About one third of all rated physicians on jameda were female. This is consistent with both the gender composition of physicians in Germany (female national average 40% [38]) and with the results by Gao and colleagues [27]. If the ratings are analyzed according to the medical specialty in relative terms (ie, compared to the national physician composition), the numbers are again confirmed by other study results. For example, Gao and colleagues showed that rated physicians were most likely to be classified as obstetrician/gynecologists and least likely to be classified as other specialists such as radiologists or anesthesiologists [27].

In this study, almost 80% of all evaluations could be assigned to the two best rating categories. Less than 3% of the physicians were rated with the worst score, “insufficient”. These results are in line with most other studies: Lagu and colleagues categorized 88% of quantitative reviews as positive, 6% as negative, and 6% as neutral [11]. On RateMDs, 45.80% of the physicians received the best score and only 12% were rated with the worst score [27]. Kadry et al assessed the 10 most commonly visited US PRWs and found that the percentage of reviews rated ≥75 on a 100-point scale was 61.5%, ≥4 on a 5-point scale was 57.74%, and ≥3 on a 4-point scale was 74.0% [32]. On the Canadian PRW RateMDs, 70% of the comments were reported to be favorable and about 30% of the comments were negative [41]. In the sample of 500 randomly selected US urologists, 86% had positive ratings [40]. Moreover, the median result of all questions in this study was “very good”. The means varied between 1.68 concerning the friendliness of the physician (question 5) and 1.85 regarding the relationship of trust with the physician (question 3). In their study, Kadry et al determined the average rating to be 77 out of 100 for sites using a 100-point scale, 3.84 out of 5 for sites using a 5-point scale, and 3.1 out of 4 for sites using a 4-point scale [32]. For the US RateMDs, the mean scores were reported to be 3.93 [27] and 3.82 [30] on a 5-point scale, respectively. Finally, a comprehensive analysis of German PRWs showed the mean ratings to be between 1.1 and 1.5 (3-point scale, 1 “good”, 3 “poor”) [34].

The results of this study suggest that female physicians receive better ratings than do their male colleagues. The number is small but statistically significant (*P*<.001). Better ratings for female physicians were also determined by Ellimoottil and colleagues (*P*=.72) [40]. However, this is in contrast to the results obtained by Gao and colleagues, who showed that male physicians received higher ratings than did female physicians (*P*<.001) [27]. But, differences in all three studies were shown to be quite small.

We can further demonstrate significant rating differences among the analyzed medical specialties. Of these, the best rated were laboratory specialists, anesthetists, medical practitioners without specialization, and family physician/general practitioners. The lowest ratings were given to neurologist/psychiatrists, ophthalmologists, orthopedists, and dermatologists. In line with the numbers obtained in this study, higher ratings were shown for physicians in primary care [27] and lower ratings for physicians in dermatology [30]. However, in another study, primary care physicians were rated at average [30]. Lagu et al found a similar percentage of positive, negative, and neutral quantitative reviews for generalists and subspecialists. They then concluded that after accounting for varying number of reviews per physician, generalists tended to have more positive reviews than did subspecialists [11].

This is the first study that allows for a closer analysis of the patients who rate their physicians. Approximately 73% of all patients provided information regarding gender, age, and health insurance. According to our results, most of the rating patients were female (60%) and were covered by Statutory Health Insurance (83%). One other notable fact could be shown: patients in the youngest age group (<30) made fewer ratings than did older patients. Whether or not this is due to more severe illness problems with increasing age cannot be assessed with this data. However, this question should be addressed in future research.

The fact that hardly any patients leave more than a single rating (mean 1.19 rated) can be regarded as even more surprising. One might expect that once they were aware of the existence of such websites, patients would use them constantly in an active (ie, rating physicians) or passive (ie, only searching for physicians) manner, especially to assist other patients with information when seeking a physician. However, we could not investigate the motivation behind the patients’ ratings. Nor could we assess the reasons for not regularly rating physicians. Considering the mean of 14 [42] to 17 [43] physician contacts in Germans with statutory health insurance, there is still high potential for even more ratings. The fact that patients covered by private health insurance give more favorable ratings than do patients covered by statutory health insurance is not surprising, since they were found to have faster access to care [44]. This might well have had an effect on the ratings differences. Whether quality of care differences can be determined between the two groups and whether this leads to ratings differences should be addressed in future studies.

It could be shown that there is a significant correlation between the mean overall performance rating of a physician and the number of ratings received for that physician (*P*<.001). One possible explanation for this finding might be the fact that physicians who are aware of these websites and use them as a marketing instrument may specifically ask satisfied patients to leave a (positive) rating on a PRW. Another explanation might be that some physicians, who are identified by patients on PRWs, simply provide outstanding quality of care and they receive favorable ratings afterwards. Although our results prove that there is a significant correlation between these variables, we cannot prove which assumption is true. This should be addressed in further studies, which should contain additional information about the physicians.

### Limitations

There are some limitations that have to be taken into account when interpreting the results of this investigation. First, we analyzed online ratings from only a single PRW, jameda. Although jameda has shown to be the most frequently used German PRW, it is possible that other PRWs have more online reviews or show other results. Second, the data provided allowed for comprehensive analysis. However, there was no information available on the age of the physician, malpractice claims, or the medical school attended. This information would have allowed further analysis. Third, we were not able to present analysis conducted over a longer period of time. However, the data do reflect the entire year 2012. Fourth, we did not analyze results presented in narrative comments. Finally, there was no chance to verify the validity of the analyzed reviews. Therefore, it cannot be guaranteed that the ratings were not subject to manipulation [27].

### Conclusions

Finally, it can be stated that there is a limited amount of publicly reported information on quality of health care providers. To increase transparency, different approaches have been developed. There are traditional PR instruments that focus on the adherence to evidence-based guidelines. Thus, they may have the potential to reflect the clinical quality of care provided by a health care professional. However, these instruments have not yet proven to be a meaningful measure for patients. In contrast, PRWs concentrate on patient satisfaction measures. Whether or not these results have the potential to reflect the quality of care provided by a health care professional should be addressed in future research as well. Since an increasing usage of these websites has already been shown [24,27,28], PRWs might contribute to reducing the lack of publicly available information on quality, at least for those physicians who have been rated. Given that only a certain number of physicians has been rated so far, there is still no perfect transparency. However, given the increasing number of ratings on PRWs, the future impact for patients seeking a physician will continue to rise.

## Abbreviations

PR: public reporting
PRW: physician rating website

## References

[1] Lebrun LA, Shi L, Zhu J, Sharma R, Sripipatana A, Hayashi AS, Daly CA, Ngo-Metzger Q (2013). Racial/ethnic differences in clinical quality performance among health centers. J Ambul Care Manage.

[2] McGlynn EA, Asch SM, Adams J, Keesey J, Hicks J, DeCristofaro A, Kerr EA (2003). The quality of health care delivered to adults in the United States. N Engl J Med.

[3] Asch SM, Kerr EA, Keesey J, Adams JL, Setodji CM, Malik S, McGlynn EA (2006). Who is at greatest risk for receiving poor-quality health care?. N Engl J Med.

[4] Laschet H (2013). ÄrzteZeitung.

[5] Dynan L, Goudie A, Smith RB, Fairbrother G, Simpson LA (2013). Differences in quality of care among non-safety-net, safety-net, and children's hospitals. Pediatrics.

[6] Merchant RM, Yang L, Becker LB, Berg RA, Nadkarni V, Nichol G, Carr BG, Mitra N, Bradley SM, Abella BS, Groeneveld PW, American Heart Association Get With the Guideline-Resuscitation Investigators (2012). Variability in case-mix adjusted in-hospital cardiac arrest rates. Med Care.

[7] Tsai C, Sullivan AF, Gordon JA, Kaushal R, Magid DJ, Blumenthal D, Camargo CA (2012). Racial/ethnic differences in emergency care for joint dislocation in 53 US EDs. Am J Emerg Med.

[8] Hibbard J, Sofaer S (2010). Best Practices in Public Reporting No 1: How To Effectively Present Health Care Performance Data To Consumers. AHRQ Publications No. 10-0082-EF.

[9] Hibbard JH (2008). What can we say about the impact of public reporting? Inconsistent execution yields variable results. Ann Intern Med.

[10] Porter ME, Guth C (2012). Chancen für das deutsche Gesundheitssystem.

[11] Lagu T, Hannon NS, Rothberg MB, Lindenauer PK (2010). Patients' evaluations of health care providers in the era of social networking: an analysis of physician-rating websites. J Gen Intern Med.

[12] Faber M, Bosch M, Wollersheim H, Leatherman S, Grol R (2009). Public reporting in health care: how do consumers use quality-of-care information? A systematic review. Med Care.

[13] Emmert M, Gemza R, Schöffski O, Sohn S (2012). [Public reporting in health care: the impact of publicly reported quality data on patient steerage]. Gesundheitswesen.

[14] Fung CH, Lim YW, Mattke S, Damberg C, Shekelle PG (2008). Systematic review: the evidence that publishing patient care performance data improves quality of care. Ann Intern Med.

[15] Mukamel DB, Mushlin AI (1998). Quality of care information makes a difference: an analysis of market share and price changes after publication of the New York State Cardiac Surgery Mortality Reports. Med Care.

[16] Chassin MR (2002). Achieving and sustaining improved quality: lessons from New York State and cardiac surgery. Health Aff (Millwood).

[17] Mukamel DB, Weimer DL, Zwanziger J, Gorthy SF, Mushlin AI (2004). Quality report cards, selection of cardiac surgeons, and racial disparities: a study of the publication of the New York State Cardiac Surgery Reports. Inquiry.

[18] Stevenson DG (2006). Is a public reporting approach appropriate for nursing home care?. J Health Polit Policy Law.

[19] NHS (2013). Quality and Outcomes Framework.

[20] NHS Choices (2013). Your health, your choices.

[21] Wubker A, Sauerland D, Wubker A (2010). Beeinflussen bessere Qualitatsinformationen die Krankenhauswahl in Deutschland? Eine empirische Untersuchung (Does Better Quality Information Affect Hospital Choice in Germany? An Empirical Analysis). Jahrbuecher fur Nationaloekonomie und Statistik.

[22] Geraedts M, Schwartze D, Molzahn T (2007). Hospital quality reports in Germany: patient and physician opinion of the reported quality indicators. BMC Health Serv Res.

[23] Hibbard JH, Peters E (2003). Supporting informed consumer health care decisions: data presentation approaches that facilitate the use of information in choice. Annu Rev Public Health.

[24] Emmert M, Sander U, Esslinger AS, Maryschok M, Schöffski O (2012). Public reporting in Germany: the content of physician rating websites. Methods Inf Med.

[25] Emmert M, Sander U, Pisch F (2013). Eight questions about physician-rating websites: a systematic review. J Med Internet Res.

[26] Hardey M (2010). Consuming Professions: User-review websites and health services. Journal of Consumer Culture.

[27] Gao GG, McCullough JS, Agarwal R, Jha AK (2012). A changing landscape of physician quality reporting: analysis of patients' online ratings of their physicians over a 5-year period. J Med Internet Res.

[28] López A, Detz A, Ratanawongsa N, Sarkar U (2012). What patients say about their doctors online: a qualitative content analysis. J Gen Intern Med.

[29] Alemi F, Torii M, Clementz L, Aron DC (2012). Feasibility of real-time satisfaction surveys through automated analysis of patients' unstructured comments and sentiments. Qual Manag Health Care.

[30] Black EW, Thompson LA, Saliba H, Dawson K, Black NM (2009). An analysis of healthcare providers' online ratings. Inform Prim Care.

[31] Emmert M, Maryschok M, Eisenreich S, Schöffski O (2009). [Websites to assess quality of care--appropriate to identify good physicians?]. Gesundheitswesen.

[32] Kadry B, Chu LF, Kadry B, Gammas D, Macario A (2011). Analysis of 4999 online physician ratings indicates that most patients give physicians a favorable rating. J Med Internet Res.

[33] Mostaghimi A, Crotty BH, Landon BE (2010). The availability and nature of physician information on the internet. J Gen Intern Med.

[34] Strech D, Reimann S (2012). Deutschsprachige Arztbewertungsportale: Der Status quo ihrer Bewertungskriterien, Bewertungstendenzen und Nutzung: German Language Physician Rating Sites: The Status Quo of Evaluation Criteria, Evaluation Tendencies and Utilization. Gesundheitswesen.

[35] Geraedts M, Hermeling P, de Cruppé W (2012). Communicating quality of care information to physicians: a study of eight presentation formats. Patient Educ Couns.

[36] Reimann S, Strech D (2010). The representation of patient experience and satisfaction in physician rating sites. A criteria-based analysis of English- and German-language sites. BMC Health Serv Res.

[37] Emmert M, Sander U, Maryschok M, Esslinger AS, Schöffski O (2010). Arzt-Bewertungsportale im Internet: Eine aktuelle Bestandsaufnahme. IMPLICONplus - Gesundheitspolitische Analysen.

[38] Bundesärztekammer (2011). Ergebnisse der Ärztestatistik zum 31.

[39] Kassenärztliche Bundesvereinigung, National Association of Statutory Health Insurance Physicians (2012). Statistische Informationen aus dem Bundesarztregister: Bundesgebiet insgesamt.

[40] Ellimoottil C, Hart A, Greco K, Quek ML, Farooq A (2013). Online reviews of 500 urologists. J Urol.

[41] Mackay B (2007). RateMDs.com nets ire of Canadian physicians. CMAJ.

[42] Grobe T, Bitzer E, Schwartz F (2013). Barmer GEK Arztreport 2013: Schwerpunkt.

[43] Riens B, Erhard M, Mangiapane S (2012). Primary care physician contacts in 2007 &amp;#8211; Background and analyses.

[44] Roll K, Stargardt T, Schreyögg J (2011). Effect of Type of Insurance and Income on Waiting Time for Outpatient Care. Working Papers, Hamburg Center for Health Economics, University of Hamburg.

